# Demographic and Clinical Characteristics of Pediculosis-associated Severe Anemia in the Emergency Department

**DOI:** 10.5811/westjem.42507

**Published:** 2025-10-21

**Authors:** William Plowe, Reed Colling, Sanjay Mohan, Rajneesh Gulati, Rana Biary, Evan Yanni, Christian A. Koziatek

**Affiliations:** * New York University Grossman School of Medicine, Ronald O. Perelman, Department of Emergency Medicine, New York, New York; † New York City Health and Hospitals Corporation, Bellevue Hospital Center, Department of Emergency Medicine, New York, New York; ‡ New York University Grossman Long Island School of Medicine, Department of Emergency Medicine, Mineola, New York

## Abstract

**Introduction:**

Infestation with Pediculus species, or common lice, is frequently diagnosed in the emergency department (ED). Because lice ingest human blood, prolonged and heavy infestation can plausibly lead to iron deficiency anemia. Severe anemia attributable to lice infestation has infrequently been reported to date. Our objective in this study was to retrospectively review cases of lice-related anemia at a single public hospital to identify risk factors and associated demographic and clinical features of this disease process.

**Methods:**

We screened the medical records for patients presenting to the ED of an urban public hospital between 2016–2024 for the diagnoses of lice infestation and severe anemia (hemoglobin < 7 grams per deciliter (g/dL). Cases were reviewed for clinical and demographic characteristics.

**Results:**

A total of 932 patients were diagnosed with pediculosis infestation in the ED during the study period; 332 (35.6%) of those patients had a complete blood count obtained by the treating team. Thirty-seven cases of severe anemia were identified (3.9% of total pediculosis cases, 11.1% of those for whom a complete blood count was obtained); 84% were microcytic, indicating iron deficiency anemia. Twenty-five patients (68%) were undomiciled, and nine patients (24%) were shelter domiciled. Twenty-three patients (62%) had comorbid psychiatric diagnoses, and 21 (51%) had substance use disorders. The median hemoglobin was 4.4 g/dL (range 2.4–6.9 g/dL). Thirty patients (81%) were admitted to a medical floor and seven patients (19%) to an intensive care unit, each with a comorbid primary condition.

**Conclusion:**

In this cohort, anemia secondary to lice infestation was seen in patients with unstable housing, substance use disorders, and psychiatric disease. Most patients were hemodynamically stable, consistent with the proposed mechanism of chronic blood loss. The prevalence of this condition may be higher than previously noted among this vulnerable population. Emergency physicians should be aware of this rare but potentially serious disease process.

## INTRODUCTION

*Pediculus humanus capitis* is a parasitic arthropod that lives on the human scalp and feeds on the host’s blood.^1^ It is closely related to *P humanus corporis*, or body lice, which lives on fomites and moves to the body for meals.^1^ Typically, lice infestation causes pruritus as a result of a localized allergic reaction to the insect’s saliva. Although most cases are mild and can be managed via anti-parasitic medications, systemic illness such as trench fever (*Bartonella quintana*), relapsing fever (*Borelia recurrentis*), and epidemic typhus (*Rickettsia prowazekii)* can ensue.^1^

Lice consume human blood for nutrition, with a typical meal being 0.008 mL per louse.^2^ In cases of mild infestation with dozens of lice, this typically represents a clinically insignificant volume of blood loss of 0.7–2.3 mL per day. There are, however, reports in the literature of chronic anemia attributed to severe, longer term lice infestation with prolonged blood loss. There are multiple case reports and a single case series of five adult patients with anemia attributed to severe lice infestation.^3^–^8^ Moreover, several pediatric cases of anemia caused by severe lice infestation in the context of neglect have been reported.^9^–^12^ The veterinary literature has also established severe species-specific lice infestation as a cause of anemia in large mammals (cattle) and spider monkeys.^13^–^18^

While a causal link has not been established, social determinants of health (ie, limited access to food; neglect; unstable housing) and associated psychiatric pathology that limit the ability to care for oneself are frequently present in published cases of severe anemia associated with lice infestation. The association between housing status, psychiatric disease, and lice infestation has been previously demonstrated.^19^ To date, no large population-based studies have been performed to further quantify the prevalence of severe lice infestation and associated risk factors for concomitant anemia or to describe the clinical characteristics of patients presenting with pediculosis-associated anemia.

## METHODS

### Study Setting and Population

This was a retrospective study of patients presenting to the emergency department (ED) at a single, urban, public ED between January 1, 2016–January 1, 2024. The ED sees an annual volume of about 110,000 patients per year and is staffed primarily by residents and attending physicians who are board-certified in emergency medicine. A large proportion of patients come from a medically underserved population including a significant unhoused population. The study was approved by the institutional review board of the affiliated school of medicine.

### Study Design and Data Analysis

We performed a retrospective, observational study of adult patients presenting with both lice infestation and anemia severe enough to warrant admission and blood transfusion. Inclusion criteria were the clinical diagnosis of lice infestation, made by direct visual identification of characteristic organisms on the patient by the treating emergency physician, and severe anemia requiring transfusion and admission—defined as a hemoglobin < 7 grams per deciliter (g/dL) during the ED encounter. We queried electronic health record (EHR) data from the Epic Clarity database (Epic Systems Corporation, Verona, WI) using Oracle SQL Developer (Oracle Corporation, Nashville, TN), and we then reviewed candidate charts to ensure they met the above inclusion criteria. We excluded patients < 18 years of age.

**Population Health Research Capsule**

What do we already know about this issue?

*Lice infestation is common in vulnerable populations and has been reported in rare cases to cause iron deficiency anemia.*

What was the research question?

*What are the prevalence, clinical, and demographic features of lice-related severe anemia in emergency department patients?*

What was the major finding of the study?

*3.9% of lice-infested patients had severe anemia requiring hospitalization (37 cases); 84% were microcytic. The median hemoglobin was 4.4 g/dL.*

How does this improve population health?

*Lice-associated severe anemia requiring hospitalization may be more common than previously reported, especially in vulnerable patient populations.*

Charts were reviewed manually to extract patient demographic data including age, sex, race, housing status, past medical history, comorbid psychiatric pathology, history of gastrointestinal (GI) bleeding, anticoagulation history, substance use disorders, and medications, as well as clinical characteristics including presenting vital signs, chief complaint on arrival to the ED, number of units of red blood cells transfused, disposition level of care from the ED, length of inpatient stay, gastroenterology consultation (if obtained), and discharge destination from inpatient stay. We also extracted relevant initial laboratory values including hemoglobin, hematocrit, mean corpuscular volume, platelets, total iron, total iron-binding capacity, ferritin, folate level, B12 level, lactate dehydrogenase, albumin, international normalized ratio, partial thromboplastin time, and bicarbonate. Charts were reviewed by four emergency physicians; elements of optimal chart review were followed, including the following: Abstractors were trained prior to review; we defined case selection criteria and variables of interest; a data abstraction form was used; and missing data elements (ie, if a lab was not obtained) were recorded. For practical reasons, abstractors were not blinded to the study hypothesis. Inter-rater reliability was not formally calculated, but charts were re-reviewed by a senior author to assure accuracy.^20^

We calculated descriptive statistics to analyze the dataset using Microsoft Excel (Microsoft Corporation, Redmond, WA). Median hemoglobin values for patients admitted to the floor vs intensive care unit were compared using a Mann-Whitney U test. Using the Kruskal-Wallis test, we compared median length of stay (LOS) for patients who left the hospital against medical advice, were discharged from the medicine service, or were transferred to inpatient psychiatry. *P*-values < .05 were considered statistically significant. We followed Strengthening the Reporting of Observational Studies in Epidemiology guidelines for this retrospective study.^21^

## RESULTS

A total of 932 patients were diagnosed with pediculosis infestation in the ED during the study period; of those, 332 had a complete blood count ordered for any reason by the treating team. A total of 37 cases met the study inclusion criteria during the study period. Thirty patients (81%) were male, and seven (19%) were female. The average age was 54.7 years of age (range 26–71). Twenty-five patients (68%) were undomiciled, and nine patients (24%) were shelter domiciled. Twenty-three patients (62%) had comorbid psychiatric diagnoses, and 21 patients (57%) had concurrent substance use disorders. Two patients (5%) had a prior documented history of GI bleeding, and no patients were on any form of anticoagulation. The full demographic data for the studied patients are detailed in Table 1.

**Figure 1:**
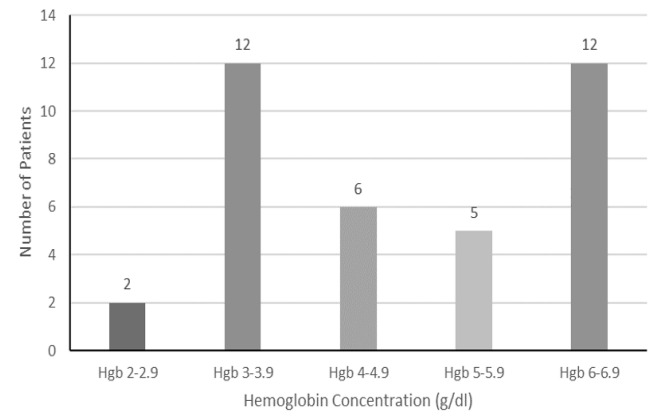
Distribution of hemoglobin concentration in patients with pediculosis-associated severe anemia. *g/dL*, grams per deciliter; *Hgb*, hemoglobin.

The mean hemoglobin was 4.79 g/dL (range 2.4–6.9 g/dL). The average mean corpuscular volume (MCV) was 73.55 fluid ounces (fL). Only one patient had an MCV > 100 fL (102 fL); 31 patients (84%) were diagnosed with microcytic anemia, defined as an MCV < 80 fL, and five patients (14%) were normocytic, defined as a MCV between 80–100 fL. Figure 1 visually delineates the severity of anemia across the full patient cohort.

The mean systolic and diastolic blood pressures on presentation were 121 and 67 millimeters of mercury (mm Hg), respectively. Eight patients (22%) were hypertensive with a systolic pressure > 135 mm Hg. Three patients (8%) were hypotensive with a systolic pressure < 90 mm Hg. The remaining 26 patients (70%) were normotensive. The presenting mean heart rate was 92 beats per minute (bpm). Thirteen patients (35%) had a heart rate > 100 bpm, two patients (5%) were bradycardic to 53 and 55 bpm, and 24 patients (65%) had a normal heart rate between 60–100 bpm.

**Figure 2:**
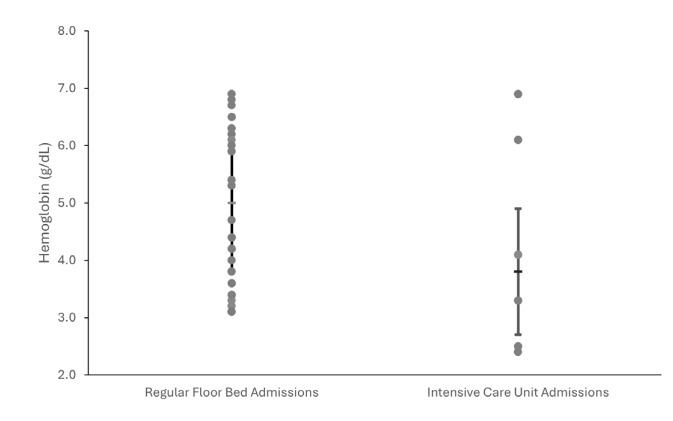
Scatter dot plot of hemoglobin values for patients admitted to the hospital for pediculosis-associated anemia to regular floor beds compared to intensive care unit beds, with median and interquartile ranges displayed via error bars. The difference in median hemoglobin between the two groups was not significant (P = .23).

Seven patients (19%) were admitted to the intensive care unit (ICU) from the ED; the remainder of the patients were admitted to a regular floor bed. Of the seven patients admitted to the ICU, six had a comorbid, primary indication for ICU level of care: four for sepsis/septic shock; and two for environmental hypothermia. The final patient was admitted to the ICU briefly for hypotension; after blood transfusion, his blood pressure improved, and he was transferred back to a regular bed within 24 hours. The median hemoglobin for floor admissions was 5 g/dL, and the median hemoglobin for ICU admissions was 3.8 g/dL. This difference was not significant (*P* = .23). Figure 2 shows the distribution of hemoglobin levels by floor- vs ICU-admitted patients.

Gastroenterology consultation was obtained during inpatient hospitalization in 11 patients (30%) to evaluate for the possibility of GI bleeding. Although endoscopy was recommended in seven of these patients, five refused, and only two underwent luminal endoscopy, each without finding evidence of acute GI bleeding. The remaining 26 patients (70%) did not receive GI consultation due to a lack of clinical concern for acute GI bleeding. No patients in the cohort had any overt clinical signs of GI bleeding, such as bright red blood per rectum, melena, or hematemesis during their hospitalization.

**Figure 3:**
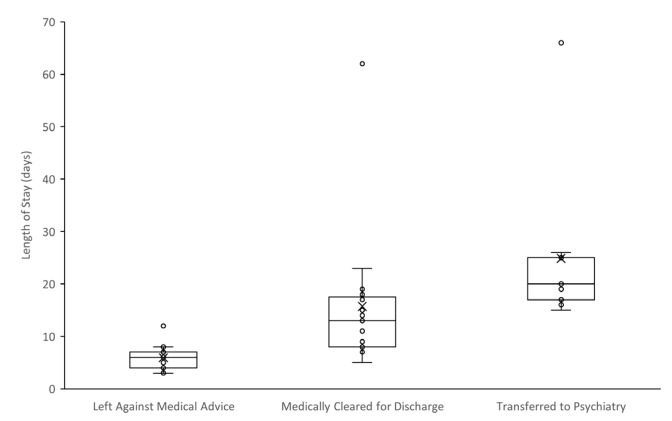
Median and box-and-whisker plot of hospital length of stay on medical service for patients admitted with pediculosis-associated severe anemia, by hospital disposition.

Thirteen patients (35%) left the hospital against medical advice, with a median LOS of six days. Fifteen patients (41%) were medically cleared for discharge, with a median LOS of 13 days. Nine patients (24%) were transferred to an inpatient psychiatry floor at the end of their medical hospitalizations, with a median LOS on the medicine service of 19 days. There was a statistically significant difference between each group (*P* < .01). Figure 3 illustrates the disposition and average LOS on the medical service for each of these groups. Notably, no patients died during their hospital course.

Additional patient specific clinical data are presented in Table 2.

## DISCUSSION

While anemia can often be multifactorial in nature, several aspects of this study group should be highlighted that point to pediculosis as a primary contributor to blood loss and resulting anemia. No clear alternate cause for anemia was found in any patient. No patients had any overt signs of GI bleeding, and the patients who did receive endoscopies had no evidence of bleeding. Only one patient had a history of chronic kidney disease, another common etiology of chronic anemia; however, it was not end stage, when severe anemia is typically seen. While chronic alcohol use disorder was common, it is typically associated with a macrocytic anemia, which was observed in only one patient in the study population (patient 3). Two female patients were premenopausal and theoretically could have had anemia due to menorrhagia, but no history of such was documented. Malnutrition may be a contributing factor to anemia and is frequently comorbid with homelessness, but it is unlikely to explain the degree of anemia observed in this cohort.^22^,^23^

Length of stay varied widely between patients who left the hospital against medical advice, those who were discharged to the community, and those transferred to an inpatient psychiatric unit at the end of their medical hospitalization. Patients requiring inpatient psychiatric hospitalization had significantly longer LOS than the other two groups, but all patient groups had substantial LOS in hospital. The psychiatric and social comorbidities we found associated with this disease process are likely significant contributors to the prolonged LOS observed in the cohort. The presence of significant psychiatric disease has previously been associated with longer LOS for somatic disease than similarly ill patients without comorbid psychiatric disease.^24^ These results suggest the value of preventative care, access to psychiatric care, and post-discharge support for these vulnerable populations in terms of preventing potentially lengthy, avoidable admissions.

Hemodynamic instability was rare in the cohort. Only two patients presented with hypotension, coincidentally both with a hemoglobin of 3.8 g/dL. More severe anemia was not associated with admission to the ICU, and all but one patient admitted to the ICU had an alternative primary pathology—either sepsis or severe environmental hypothermia. These findings are consistent with the proposed mechanism of chronic blood loss that allows for physiologic compensation for the anemia. In previously reported cases, hypotension was slightly more frequent; when blood pressure was reported, hypotension was seen in two of eight adults and two of three children.^4^–^6^,^8^–^10^,^12^ However, in the case series that most parallels ours by Guss et al, which described five homeless patients with alcohol use disorder and anemia from lice infestation, all five patients were normotensive.^6^

When analyzing the demographics of this cohort, profound anemia associated with lice infestation occurred almost exclusively in patients who had unstable housing, psychiatric disease leading to an inability to care for oneself, or both. Schizophrenia, in particular, was diagnosed in 16 patients (43%), despite being found in just 0.25–0.46% of the United States population.^25^ Diagnostic criteria for schizophrenia include a loss of the ability to care for oneself as a result of disorganized behavior, as was observed in this cohort.^26^ As is seen in other diseases, schizophrenia and severe mental illness appear to be associated with worsened outcomes compared to the general population suffering from lice infestations.^27^ These findings are consistent with the previously reported cases in adults where either substance use, psychiatric disease, or both, were noted.

Most of the adult patients in those cases also lacked housing. One patient was housed but suffered from severe depression, and the other was psychiatrically ill but taken care of by family members. In three pediatric case reports, either extreme poverty or neglect were noted leading to a delay in treatment for lice infestation.^9^,^10^,^12^ Similarly, in this cohort, all but three patients were either in the shelter system or undomiciled. Of those three patients who did have housing, two were later diagnosed with schizophrenia and the third with dementia.

The common thread among these cases is an inability to engage in sufficient self-care prior to the development of severe anemia. In this cohort, patients with substance use disorders, unstable housing, and psychiatric disease either did not or could not seek timely medical care, leading to the development of severe chronic anemia. Social determinants of health such as these are frequently a major contributor to medical illness.^28^ In unhoused populations, a number of factors increase the risk of lice infestation and may worsen the severity and/or duration of infestation: inability to reliably launder clothing and bedding; lack of access to hygiene and shower facilities; and frequently communal shelter arrangements that can facilitate transmission of infectious disease.^29^–^32^ Coupled with barriers to accessing routine medical care, and substance use and psychiatric comorbidities, these patients are uniquely vulnerable to severe and prolonged infestations that can cause a chronic severe anemia. The ED often serves as the “safety net” for these patients, providing the primary point of contact for undomiciled patients within the healthcare system.^33^ Other primary medical processes that could contribute to inability to fully care for self were also observed in the cohort. Blindness, cognitive impairment, dementia, and primary stroke were also noted in a handful of patients.

The association of this disease process with lack of housing is of particular importance. In New York City, over 80,000 people are experiencing homelessness, the most of any city in the US.^34^ Emergency departments serve as the primary access to healthcare for these patients, but they frequently experience bias from clinicians despite being at greater risk for serious injury and illness than the general population.^35^,^36^ Patients with lice infestation may experience further bias and avoidance from clinicians due to the visible nature of the disease and resulting fear of transmission to staff. The typical treatment for severe lice infestation is delousing, which is often done before any further medical evaluation or admittance into the ED. The data presented here suggest that patients with severe infestations may have significant anemia that warrants urgent investigation.

The cohort reviewed here exceeds the total number of reported cases in the literature and were found over an eight-year period at a single center, suggesting a higher prevalence of lice-associated severe anemia than has previously been recognized. While the etiology of the severe anemia observed in this cohort may be multifactorial—in some cases beyond the possible causes assessed in this study—there exists a plausible mechanism by vulnerable groups with severe deficits in self-care that may present with a primarily lice-mediated anemia. Emergency physicians should be aware of the possibility of severe anemia in undomiciled patients or patients with severe psychiatric disease presenting with severe lice infestations.

## LIMITATIONS

There are several important limitations to this study. Given the retrospective nature of this project, it would be incorrect to assume that lice infestation alone was the sole etiology of anemia. Malnutrition and alcohol use disorder are common in patients with psychiatric disease and likely contributed to anemia in many cases. We could not assess for the possibility of pre-existing anemia from other possible causes (eg, as a side effect of psychiatric medication). Moreover, the diagnosis of lice infestation in each case was made clinically by the emergency physician without definitive entomological identification of the organism found on the patient.

Additionally, most patients did not receive upper and lower endoscopies to definitively rule out an occult GI hemorrhage, although no patient included in the series had clinical evidence of overt bleeding and GI bleeding was clinically ruled out in most cases. Lastly, it is unknown how many patients with severe lice infestation were deloused with no further lab tests performed and, thus, were not included in this study. It is likely that lice infestation causing severe anemia meeting transfusion criteria is underdiagnosed, and cases are often missed.

## CONCLUSION

This study suggests that patients with unstable housing, comorbid psychiatric disease, and/or substance use disorders who present with lice infestation may have severe anemia despite apparent hemodynamic stability. The prevalence of this condition may be higher than previously described, especially among these vulnerable populations. Future research directions should include prospective studies that help to further elucidate the prevalence and characteristics of lice-associated anemia.

## Figures and Tables

**Table 1:** Demographic characteristics of patients diagnosed with pediculosis-associated severe anemia (n = 37).

Yearly Mean Age (Range)	54.7 (26 – 71)
Sex (%)	
Male	30 (81%)
Female	7 (19%)
Race (%)	
White	11 (30%)
Black	18 (49%)
Hispanic	7 (19%)
Asian	1 (2%)
Housing Status (%)	
Undomiciled	25 (68%)
Shelter domiciled	9 (24%)
Domiciled	3 (8%)
Psychiatric Disease (%)	
Schizophrenia	16 (43%)
Bipolar	2 (5%)
Personality disorder	5 (14%)
None	14 (38%)
Substance Use Disorder (%)	
Yes	21 (57%)
No	16 (43%)
History of Gastrointestinal Bleeding (%)	
Yes	2 (5%)
No	35 (95%)
History of Chronic Kidney Disease (%)	
Yes	1 (3%)
No	36 (97%)
Anticoagulation	
Yes	0 (0%)
No	37 (100%)

**Table 2:** Case-specific demographic and clinical characteristics of patients with pediculosis-associated severe anemia.

Patient	Age (years)	Sex	Ethnicity	Housing Status	Psychiatric Diagnoses	Comorbid Medical Diagnoses	Substance Use Disorder	Presenting Blood Pressure (mm Hg)	Presenting Heart Rate (beats/minute)	Hemoglobin (g/dL)	Admission Destination	Mean Corpuscular Volume (fL)	Total Iron (mcg/dL)	Ferritin (ng/mL)	Total Iron- binding Content (mcg/dL)
1	62	M	White	Undomiciled	Schizophrenia	monocular blindness	N	169/84	77	6.7	floor	65.6	16	35	308
2	65	M	Black	Shelter Domiciled	None	hypertension, asthma, dementia	N	127/70	95	3.8	floor	60.8	9	5	437
3	64	F	White	Domiciled	Schizophrenia	breast cancer, chronic kidney disease, hyperlipidemia, stroke	N	115/94	84	6.3	floor	102.3	43	47	227
4	33	M	Black	Undomiciled	Schizophrenia	none	Y	125/75	112	3.1	floor	71.3	19	17	287
5	71	M	Black	Domiciled	Personality Disorder	dementia	N	122/68	98	4.2	floor	79.1	not obtained	not obtained	not obtained
6	62	M	Hispanic	Shelter Domiciled	Personality Disorder	diabetes mellitus, stroke	N	111/47	101	3.8	floor	70.3	15	18	214
7	61	F	White	Undomiciled	None	none	N	107/54	113	3.6	floor	80.1	7	7	329
8	62	F	White	Undomiciled	Schizophrenia	hyperlipidemia	N	150/91	116	6.8	floor	71.1	11	38	280
9	68	M	White	Undomiciled	None	none	Y	99/59	104	4.7	floor	77.4	12	15	220
10	50	M	Black	Undomiciled	Schizophrenia	hypertension, coronary artery disease	N	110/50	111	3.3	floor	60	8	12	342
11	33	M	Hispanic	Undomiciled	None	none	Y	134/67	118	3.4	floor	67.4	<20	39	<20
12	58	M	White	Undomiciled	None	none	Y	87/62	53	3.3	ICU	88.1	231	14	Unable to calculate
13	65	M	Black	Undomiciled	Bipolar Disorder	none	Y	76/45	73	3.8	floor	64.7	34	12	347
14	69	F	White	Undomiciled	Schizophrenia	unspecified anemia	N	102/41	84	2.4	ICU	65.2	91	5	373
15	29	F	Black	Undomiciled	Bipolar Disorder	none	Y	119/52	120	2.5	ICU	82.9	12	12	294
16	57	M	Hispanic	Undomiciled	None	none	Y	125/83	99	6.9	floor	65.7	<20	12	<20
17	71	M	Hispanic	Undomiciled	Schizophrenia	diabetes mellitus	Y	96/51	99	6.2	floor	64.9	20	6	284
18	27	M	Black	Shelter Domiciled	Schizophrenia	seizure disorder	N	103/54	96	4	floor	59.7	11	2	443
19	26	M	Asian	Undomiciled	Schizophrenia	peripheral vascular disease	Y	106/56	119	5.4	floor	56.1	21	216	140
20	63	M	Black	Undomiciled	None	hypertension, seizure disorder	Y	128/67	82	3.2	floor	77	<20	40	<10
21	62	M	Hispanic	Shelter Domiciled	None	diabetes mellitus	Y	111/47	101	3.8	floor	70	14	18	223
22	48	M	Black	Undomiciled	Schizophrenia	lupus	N	114/57	100	5.3	floor	92	21	38	244
23	62	M	White	Shelter Domiciled	None	cirrhosis	Y	163/96	66	6.3	floor	99	12	123	127
24	33	M	Black	Undomiciled	Schizophrenia	none	N	127/76	94	3.1	floor	71	19	17	287
25	65	M	White	Undomiciled	None	none	N	85/28	77	3.8	ICU	64.7	34	12	347
26	71	F	Black	Domiciled	Schizophrenia	none	N	107/58	106	4.2	floor	79.1	68	78	267
27	49	M	Hispanic	Undomiciled	None	cirrhosis, diabetes mellitus	Y	160/109	117	5.9	floor	81	53	101	166
28	54	M	Hispanic	Undomiciled	None	cognitive impairment, pituitary macroadenoma	Y	124/76	80	6.9	ICU	88	12	197	151
29	68	M	White	Shelter Domiciled	None	diabetes mellitus, hypertension	Y	109/67	92	6.1	floor	70	11	18	382
30	61	M	Black	Shelter Domiciled	Schizophrenia	hypertension	N	134/71	91	6.3	floor	94	31	195	256
31	30	F	Black	Shelter Domiciled	Personality Disorder	none	N	103/62	75	6.5	floor	65	16	67	328
32	65	M	Black	Undomiciled	None	hypertension, peripheral vascular disease, blindness	Y	154/74	85	6.1	ICU	77	14	32	256
33	58	M	Black	Undomiciled	Personality Disorder	stroke, traumatic brain injury, seizure disorder, hypertension	Y	160/97	78	6	floor	68.9	<20	90	<20
34	30	M	Black	Undomiciled	Schizophrenia	None	Y	114/71	93	4.4	floor	75.6	12	14	286
35	65	M	Black	Undomiciled	Schizophrenia	venous thromboembolism	Y	134/84	55	4.1	ICU	63.5	7	31	218
36	53	M	White	Undomiciled	Personality Disorder	uveitis, blindness	Y	141/77	83	5.9	floor	70	not obtained	not obtained	not obtained
37	53	M	Black	Shelter Domiciled	Schizophrenia	none	Y	141/67	80	5.3	floor	62.7	6	4	415

*dL*, deciliter; *Fl*, fluid ounces; *g*, gram; *mcg*, microgram; *mm Hg*, millimeters of mercury; *ng*, nanogram.
